# Supplementary material for: Structural determinants and genetic modifications enhance BMP2 stability and extracellular secretion
Source: FASEB Bioadv. 2019 Jan 2;1(3):180–90. doi: 10.1096/fba.2018-00023 (PMC6586023; doi:10.1096/fba.2018-00023)
Supplement: Supplementary file 1 [file FBA2-1-180-s001.pdf]

Supplementary Information

Structural determinants and genetic modifications enhance BMP2 stability and extracellular secretion

Vinayak Khattar, Joo Hyoung Lee, Hong Wang, Soniya Bastola, and Selvarangan Ponnazhagan

Supplementary Figure 1

Blocking of proteasomal degradation pathway with MG-132 leads to an increase in BMP2 level and enhances secretion

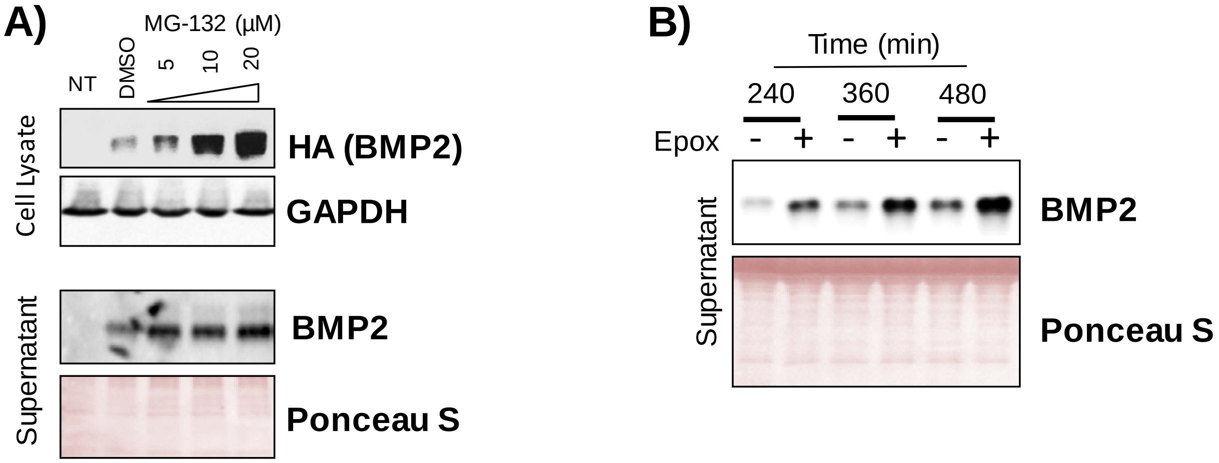

A) 293T cells were transfected with a mammalian expression vector encoding BMP2. The cells were split 24 hours post-transfection and treated with indicated dose of MG-132. The cells were harvested and media from cells was collected 6 hours post treatment. Intracellular BMP-2 level (upper panel) and secreted BMP-2 level (lower panel) was determined by immunoblotting.

B) 293T cells were transfected with pCMV3-HA-BMP2 and treated with 20 micromolar MG-132 for increasing duration of time. Overlaying conditioned media was harvested at regular intervals, and BMP2 secretion into the medium was assessed by western blotting. MG-132-devoid medium was used as a control to monitor the transfection driven increase in BMP2.

Supplementary Figure 2

Homology modeling of BMP2 depicting a lysine cluster affecting stability

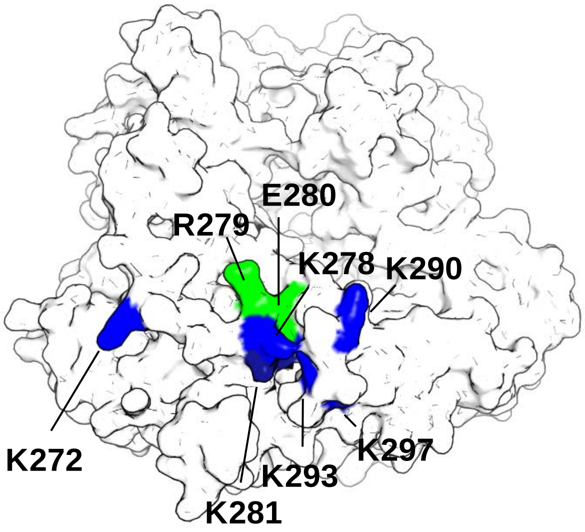

Sphere model of BMP2 showed that the indicated lysine residues (shown in blue) form a cluster adjacent to the cleavage site (shown in green) that releases mature BMP2. All structure figures are generated using PyMol

Supplementary Table 1: Primers used for construction of K-to-R mutants of BMP-2

BMP-2 Mutation	Mutagenesis Primer Sequence
K32R	F 5'-GGGCCGCAGGA**G**GTTCGCGGCGG-3'<br>R 5'-CCGCCGCGAAC**C**TCCTGCGGCCC-3'
K64R	F 5'-CTCAGCATGTTCGGCCTGA**G**ACAGAGACCC-3'<br>R 5'-GGGTCTCTGT**C**TCAGGCCGAACATGCTGAG-3'
K127R	F 5'-CCAGAAACGAGTGGGA**G**AACAACCCGGAGATTC-3'<br>R 5'-GAATCTCCGGGTTGTT**C**TCCCACTCGTTTCTGG-3'
K178R	F 5'-CATCACCGAATTAATATTTATGAAATCATAA**G**ACCTGCAACAGCCAAC-3'<br>R 5'-GTTGGCTGTTGCAGGT**C**TTATGATTTCATAAATATTAATTCGGTGATG-3'
K185R	F 5'-CAACAGCCAACTCGA**G**ATTCCCCGTGACCAG-3'<br>R 5'-CTGGTCACGGGGAAT**C**TCGAGTTGGCTGTTG-3'
K236R	F 5'-GCCCACTTGGAGGAGA**G**ACAAGGTGTCTCC-3'<br>R 5'-GGAGACACCTTGT**C**TCTCCTCCAAGTGGGC-3'
K241R	F 5'-GAGAAACAAGGTGTCTCCA**G**GAGACATGTTAGGATAAGC-3'<br>R 5'-GCTTATCCTAACATGTCTC*C*TGGAGACACCTTGTTTCTC-3'
K272R	F 5'-AGTAACTTTTGGCCATGATGGAA**G**AGGGCATCCTCT-3'<br>R 5'-AGAGGATGCCCT**C**TTCCATCATGGCCAAAAGTTACT-3'
K278R	F 5'-AGGGCATCCTCTCCACA**G**AAGAGAAAAACGTCAAG-3'<br>R 5'- CTTGACGTTTTTCTCTT**C**TGTGGAGAGGATGCCCT -3'
K281R	F 5'-GCATCCTCTCCACAAAAGAGAAA**G**ACGTCAAGCCAA-3'<br>R 5'-TTGGCTTGACGT**C**TTTCTCTTTTGTGGAGAGGATGC-3'
K285R	F 5'-AAGAGAAAAACGTCAAGCCA**G**ACACAAACAGCGGAAAC-3'<br>R 5'-GTTTCCGCTGTTTGTGT**C**TGGCTTGACGTTTTTCTCTT-3'
K287R	F 5'-AGAAAAACGTCAAGCCAAACACA**G**ACAGCGGAAACG-3'<br>R 5'-CGTTTCCGCTGT**C**TGTGTTTGGCTTGACGTTTTTCT-3'
K290R	F 5'-AAGCCAAACACAAACAGCGGA**G**ACGCCTTAAGTCC-3'<br>R 5'-GGACTTAAGGCGT**C**TCCGCTGTTTGTGTTTGGCTT-3'
K293R	F 5'-ACAGCGGAAACGCCTTA**G**GTCCAGCTGTAAGAG-3'<br>R 5'-CTCTTACAGCTGGAC**C**TAAGGCGTTTCCGCTGT-3'
K297R	F 5'-GCCTTAAGTCCAGCTGTA**G**GAGACACCCTTTGTA-3'<br>R 5'-TACAAAGGGTGTCTC**C**TACAGCTGGACTTAAGGC-3'
K355R	F 5'-GTCAACTCTGTTAACTCTA**G**GATTCCTAAGGCATGCTGT-3'<br>R 5'-ACAGCATGCCTTAGGAATC**C**TAGAGTTAACAGAGTTGAC-3'
K358R	F 5'-ACTCTGTTAACTCTAAGATTCCTA**G**GGCATGCTGTGTCC-3'<br>R 5'-GGACACAGCATGCC**C**TAGGAATCTTAGAGTTAACAGAGT-3'
K379R	F 5'-GACGAGAATGAAAAGGTTGTATTAA**G**GAACTATCAGGACATGG-3'<br>R 5'-CCATGTCCTGATAGTTC**C**TTAATACAACCTTTTCATTCTCGTC-3'
K382R	F 5'-GACGAGAATGAAAAGGTTGTATTAA**G**GAACTATCAGGACATGG-3'<br>R5'-CCATGTCCTGATAGTTC**C**TTAATACAACCTTTTCAttTTCTCGTC-3'
